# Prevalence and patterns of antifolate and chloroquine drug resistance markers in *Plasmodium vivax* across Pakistan

**DOI:** 10.1186/1475-2875-12-310

**Published:** 2013-09-05

**Authors:** Aamer A Khattak, Meera Venkatesan, Lubna Khatoon, Amed Ouattara, Leo J Kenefic, Muhammad F Nadeem, Farida Nighat, Salman A Malik, Christopher V Plowe

**Affiliations:** 1 Department of Biochemistry, Faculty of Biological Sciences, Quaid-i-Azam University, Islamabad, Pakistan; 2 Howard Hughes Medical Institute/Center for Vaccine Development, University of Maryland School of Medicine, Baltimore, MD, USA; 3 Worldwide Antimalarial Resistance Network Molecular Module, University of Maryland School of Medicine, Baltimore, MD, USA; 4 Department of Medical and Research Technology, University of Maryland School of Medicine, Baltimore, MD, USA; 5 King Edward Medical University, Lahore, Pakistan; 6 Department of Biochemistry and Molecular Biology, University of Gujrat, Gujrat, Pakistan; 7 Islamic International Medical College, Rawalpindi, Pakistan

**Keywords:** *Plasmodium vivax*, Malaria, Pakistan, Drug resistance, Sulphadoxine-pyrimethamine, Chloroquine, *Pvmdr1*, *Pvdhfr*, *Pvdhps*

## Abstract

**Background:**

*Plasmodium vivax* is the most prevalent malaria species in Pakistan, with a distribution that coincides with *Plasmodium falciparum* in many parts of the country. Both species are likely exposed to drug pressure from a number of anti-malarials including chloroquine, sulphadoxine-pyrimethamine (SP), and artemisinin combination therapy, yet little is known regarding the effects of drug pressure on parasite genes associated with drug resistance. The aims of this study were to determine the prevalence of polymorphisms in the SP resistance-associated genes *pvdhfr*, *pvdhps* and chloroquine resistance-associated gene *pvmdr1* in *P. vivax* isolates collected from across the country.

**Methods:**

In 2011, 801 microscopically confirmed malaria-parasite positive filter paper blood samples were collected at 14 sites representing four provinces and the capital city of Islamabad. Species-specific polymerase chain reaction (PCR) was used to identify human *Plasmodium* species infection. PCR-positive *P. vivax* isolates were subjected to sequencing of *pvdhfr*, *pvdhps* and *pvmdr1* and to real-time PCR analysis to assess *pvmdr1* copy number variation.

**Results:**

Of the 801 samples, 536 were determined to be *P. vivax*, 128 were *P. falciparum*, 43 were mixed *vivax*/*falciparum* infections and 94 were PCR-negative for *Plasmodium* infection. Of PCR-positive *P. vivax* samples, 372 were selected for sequence analysis. Seventy-six of the isolates (23%) were double mutant at positions S58R and S117N in *pvdhfr*. Additionally, two mutations at positions N50I and S93H were observed in 55 (15%) and 24 (7%) of samples, respectively. Three 18 base pair insertion-deletions (indels) were observed in *pvdhfr*, with two insertions at different nucleotide positions in 36 isolates and deletions in 10. Ninety-two percent of samples contained the *pvdhps* (S382/A383G/K512/A553/V585) SAKAV wild type haplotype. For *pvmdr1*, all isolates were wild type at position Y976F and 335 (98%) carried the mutation at codon F1076L. All isolates harboured single copies of the *pvmdr1* gene.

**Conclusions:**

The prevalence of mutations associated with SP resistance in *P. vivax* is low in Pakistan. The high prevalence of *P. vivax* mutant *pvmdr1* codon F1076L indicates that efficacy of chloroquine plus primaquine could be in danger of being compromised, but further studies are required to assess the clinical relevance of this observation. These findings will serve as a baseline for further monitoring of drug-resistant *P. vivax* malaria in Pakistan.

## Background

Malaria imposes a significant public health burden in Pakistan, causing a reported 1.6 million cases per year according to the World Health Organization (WHO) [1]. *Plasmodium vivax* is the most prevalent species of human malaria in the country, accounting for 200,000 (67%) of all cases in 2011 [2], with *Plasmodium falciparum* malaria accounting for the remaining one-third. Chloroquine plus primaquine is the first-line treatment for *P. vivax* in most of the world, including Pakistan. After the rise of chloroquine resistance in *P. falciparum*, sulphadoxine-pyrimethamine (SP) treatment became widespread throughout Asia and has since been replaced by artesunate plus SP for treatment of falciparum malaria in Pakistan. Despite its prevalence and public health importance, *P. vivax* has not been well-characterized with respect to drug resistance to chloroquine or to SP (to which it may be exposed during presumptive treatment of *P. falciparum*) in Pakistan [3].

Chloroquine resistance in *P. vivax* has been observed in the Pacific [4], Latin America [5], and parts of Asia [6,7] but has not yet been reported in Pakistan and Afghanistan [8,9], and chloroquine remains effective against *P. vivax* in India [10,11]. Chloroquine resistance in *P. vivax* is thought to be mediated by single nucleotide polymorphisms (SNPs) in the *pvmdr1* gene of *P. vivax*[12-14]. It has been suggested that point mutations at codons Y976F and F1076L of the *pvmdr1* gene may be involved in resistance to 4-aminoquinolones, chloroquine and amodiaquine [12,15]. *In vitro* data show that Indonesian *P. vivax* isolates with a prevalence of 96% for mutation Y976F had a significantly higher IC_50_ for chloroquine compared to Thai isolates, which had a 25% prevalence of Y976F [16]. Whereas an association of mutation F1076L with chloroquine resistance has not yet been found [16,17], the most common *pvmdr1* haplotypes found in nature are F1076L [18-20], and Y976F plus F1076L [21], with the single mutantY976F reported only rarely [16]. This observation has given rise to the theory that a two-step mutational trajectory, with mutations at codon F1076L followed by Y976F, may lead to chloroquine resistance [12,22]; however, *in vivo* substantiation of this theory is required. Polymorphisms in copy number of the *pvmdr1* gene may also modulate drug resistance in *P. vivax*, as shown by a link between gene amplification and decreased in vitro susceptibility to mefloquine, artesunate, and amodiaquine but increased susceptibility to chloroquine [23].

Although SP has not been indicated for treatment of *P. vivax*, there is evidence that intensive SP use for treatment of *P. falciparum* may also select for resistant *P. vivax* in regions where both species coexist [24-26]. An efficacy study of SP, chlorproguanil-dapsone and chloroquine carried out from 2004 to 2006 suggests that antifolates were effective against *P. vivax* in Afghanistan and Pakistan at that time [9]. In the absence of more recent data, molecular markers may be used to monitor the effects of SP pressure on *P. vivax*.

Pyrimethamine and sulphadoxine target the *dhfr* and *dhps* enzymes in *P. falciparum* and point mutations in these genes confer resistance to each drug [27-34]. Mutations in codons I13L, P33L, F57L/I, S58R, T61M, S117N/T and I173L/F of the P. vivax *dhfr* enzyme have been proposed as conferring similar resistance to pyrimethamine in *P. vivax*[19,35]. *In vitro* studies of *pvdhfr* expression in *Escherichia coli*, *P. falciparum* and yeast have shown that *pvdhfr* double and triple mutants S58R + S117N, F57L + S58R, and F57L + S58R + S117T confer significant (up to several hundred-fold) reductions in susceptibility to antifolates [36-39] and are associated with reduced drug affinity to the target enzyme [38]. In *P. vivax dhps*, mutations at codons S382A/C, A383G, K512M/T/E, and A553G/C are hypothesized to confer resistance to sulphadoxine [25,40].

In Pakistan, published data are not available on the prevalence of point mutations in *pvmdr1*, and limited information is available regarding the prevalence of mutations in *pvdhfr* and *pvdhps*. In 2009, a study conducted in Bannu district of Pakistan reported a prevalence of 1.7% of *pvdhfr* F57L, 19% for S58R and 93.5% for S117N [3]. In *pvdhps*, a study conducted in 2010 in the Federally Administered Tribal Areas of Khyber Pakhtunkhwa province reported that 15% of samples had A383G and 54% had A553G/C [41]. Because up to date information on clinical efficacy and molecular markers of anti-malarial resistance in Pakistan is not available, this study was designed to evaluate the current prevalence of point mutations in *pvdhfr, pvdhps* and *pvmdr1* genes of *P. vivax* in 14 sites around the country. The results presented here provide indications of whether efficacy of current anti-malarials is being maintained against *P. vivax* in Pakistan or requires further assessment.

## Methods

### Sample collection and ethics

**Figure 1:**
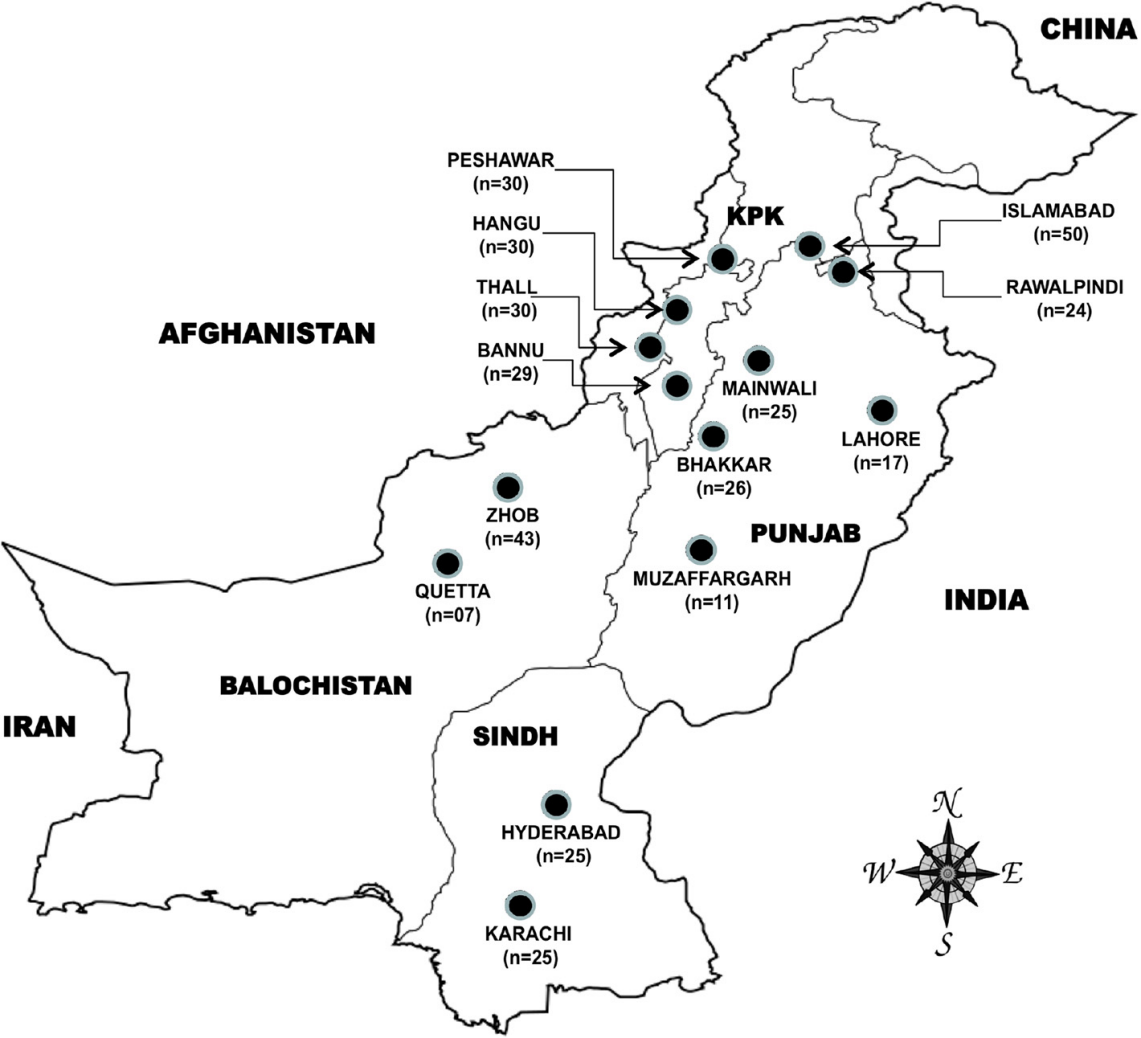
***Plasmodium vivax *collection sites and sample sizes selected for sequencing.** Total sample sizes by province were as follows: Khyber Pakhtunkhwa (119), Islamabad (50), Punjab (103), Balochistan (50) and Sindh (50).

Twenty-five government and private hospitals in the four provinces with the highest malaria burden (Khyber Pakhtunkhwa, Sindh, Balochistan and Punjab) along with the capital city were invited to provide samples collected during routine malaria surveillance. Fourteen facilities responded and were included in this study: Islamabad (the capital), Peshawar, Thall, Bannu and Hangu (Khyber Pakhtunkhwa Province; KPK), Karachi and Hyderabad (Sindh Province), Zhob and Quetta (Balochistan Province), Rawalpindi, Bhakhar, Mainwali, Lahore and Muzaffarghar (Punjab Province) (Figure 1) [42]. Despite the high prevalence of malaria, the Federally Administered Tribal Areas were excluded from this study because political uncertainty and terrorism [43] make it difficult to establish sample collection. Samples were collected between April and October 2011. A total of 10,782 symptomatic patients of all ages presenting with signs of malaria was screened for malaria infection by microscopic examination of blood by a trained laboratory technician. Approximately 3 ml of venous blood was drawn in EDTA tubes from each patient for suspected cases after obtaining informed consent. Information on patient age, gender, and city of residence was collected. The study was approved by the institutional review board of Quaid-i-Azam University, Islamabad, Pakistan.

### Microscopy and dried blood spot collection

Thick and thin blood films of patients with suspected malaria were stained with 10% Giemsa solution and slides were examined at 100X under oil immersion of microscope by laboratory technician/technologist trained in malaria diagnosis in line with WHO guidelines [44]. For samples testing positive for malaria by microscopy, 50 μl blood was applied to Whatman 3MM filter paper. Blood from 30 malaria-negative samples by microscopy was also spotted on filter paper. The remaining blood from all samples was stored on site at −80°C. Filter papers were air-dried overnight and stored in individual plastic bags with desiccant at room temperature.

### DNA extraction and speciation

DNA was isolated from 801 malaria-positive by microscopy and 30 malaria-negative filter paper samples using the QIAmp 96 DNA kit (Qiagen, Valencia, CA, USA). Isolated DNA was stored at − 20°C for further analysis. *Plasmodium* species (*P. vivax*, *P. falciparum*, *P. ovale* and *P. malariae*) were confirmed by nested polymerase chain reaction (PCR) amplification of the small subunit ribosomal ribonucleic acid (ssrRNA) genes using primers and thermal cycler conditions as previously described [45]. Twenty-seven of the 30 microscopy-negative samples were also subjected to PCR for quality control. PCR products were visualized by 2-2.5% agarose gel electrophoresis using the Bio-Rad gel doc system (Bio-Rad, Hercules, CA, USA). Molecular analysis was conducted at the University of Maryland School of Medicine, Baltimore, USA.

### Amplification of *pvdhfr, pvdhps* and *pvmdr1*

Out of 579 PCR-confirmed (536 *P. vivax* and 43 *P. vivax/P. falciparum* mixed infection) samples, 372 isolates were randomly selected for further molecular characterization (Table 1). Target sequences of the *pvdhfr*, *pvdhps* and *pvmdr1* genes harbouring known or putative mutations associated with SP and chloroquine resistance were selected for nested PCR amplification and sequencing. Primers, reagent concentrations and thermal cycling conditions were adapted from those previously published [19] with the following modifications. Denaturation was done at 94°C for 30 sec, 2 μl of DNA template was added to a total reaction volume of 50 μl, and the second round of PCR was carried out for a total of 25 cycles. PCR products were visualized as previously described. The expected band sizes for *pvdhfr*, *pvdhps* and *pvmdr1* were 632, 767 and 547 bp, respectively.

**Table 1:** **Prevalence of multilocus genotypes and single nucleotide polymorphism in *pvdhfr*, *pvdhps *and *pvmdr1 *gene of *P. vivax***

**Gene**	**Sample size**	Haplotypes*	**No of isolates (%)**
***pvdhfr***	336	IPFSTSI (wild type)	151 (45)
		IPFST**N**I	100 (30)
		IPF**R**T**N**I	76 (23)
		IP**LR**TSI	5 (1.5)
		IPF**R**TSI	3 (1.0)
		IPF**I**T**N**I	1 (0.3)
***pvdhps***	339	SAKAV (wild type)	330 (97)
		S**G**KAV	7 (2.0)
		SAK**G**V	1 (0.3)
		S**G**K**G**V	1 (0.3)
***pvdhfr + pvdhps***	322	IPFSTSISAKAV (wild type)	143 (44)
		IPFST**N**ISAKAV	95 (30)
		IPF**R**T**N**ISAKAV	69 (21)
		IP**LR**TSISAKAV	5 (1.6)
		IPF**R**TSISAKAV	3 (1.0)
		IPFST**N**IS**G**KAV	2 (0.6)
		IPF**R**T**N**IS**G**KAV	2 (0.6)
		IPFSTSISAK**G**V	1 (0.3)
		IPFSTSIS**G**KAV	1 (0.3)
		IPF**R**T**N**IS**G**K**G**V	1 (0.3)
***pvmdr1***	342	YF (wild type genotype)	7 (2.0)
		Y**L** (single mutant genotype)	335 (98)
		**F**F (single mutant genotype)	0
		F**L** (double mutant genotype)	0

*Drug resistance conferring point mutations are shown for the following codons: *pvdhfr* 13, 33, 57, 58, 61, 117, 173; *pvdhps* 382, 383, 512, 553, 585; and *pvmdr1* 976, 1076. Mutated amino acids are shown in bold.

### Sequencing

Amplified secondary PCR products of *pvdhfr*, *pvdhps* and *pvmdr1* genes were purified using Millipore® filtration plates (EMD Millipore, Billerica, MA, USA). The sequencing reaction for all three genes was performed in the forward and reverse directions using secondary PCR primers. The BigDye terminator v3.1 kit (ABI) was used for each sequencing reaction in the following ratios: BigDye 1 μl, buffers 1 μl, 2 μmol of nested PCR forward or reverse primers, purified PCR products 2 μl and ultra pure water 5 μl in a volume of 10 μl. The thermal cycler was programmed as follows: 94°C for 20 min, 25 cycles of 94°C for 20 sec, 50°C for 5 sec, 48°C for 5 sec, 60°C for 3 min, and final extension at 60°C for 5 min. Sequencing was performed using an ABI 3730XL automatic sequencer (Applied Biosystems, Foster City, CA) and sequence analyses and alignment was performed using Sequencher 4.10.1 software (Gene Codes Corp., Ann Arbor, MI, USA). Sequences of a *P. vivax* ARI/Pakistan isolate (Gen-Bank accession no. X98123), Brazilian clinical isolate (accession no AY186730) and Sal-1 (Gen-Bank accession no. AY618622) were used as references for polymorphisms in *pvdhfr*, *pvdhps* and *pvmdr1*.

### Confirmation of rare variants

Rare or novel SNP variants were ruled out as PCR or sequencing errors as follows. SNPs occurring in a single sample and those not previously reported were re-amplified by PCR and sequenced a second time to confirm presence of the polymorphism. Original and re-sequencing reactions were both conducted in the forward and reverse directions (2X coverage) to reduce the likelihood of sequencing errors.

### Quantification of *pvmdr1* copy number

Quantification of *pvmdr1* copy number was assessed using previously described primers, probes and thermal cycler conditions [19] on a TaqMan 7700 real-time PCR machine (Applied Biosystems, Warrington, UK). *Pvaldolase* was used as the endogenous control and a plasmid containing a single and double copy number insert of *pvmdr1* in pCR2.1 vector was used as calibrator. All samples were run in triplicate. *pvmdr1* copy number values between 0.5 and 1.3 were considered as single copy, between 1.6 and 2.3 as two copies and between 2.6 and 3.3 as three copies. Assays were repeated if copy number values were between 1.3 and 1.6 and 2.3 and 2.6. Assays were also repeated if the Ct value exceeded 35 or the standard deviation for replicates of *pvmdr1* or *pvaldolase* copy number exceeded 0.2. Copy number of *pvmdr1* was estimated by using the following formula:

$$\begin{array}{l} \Delta \Delta \mathrm{Ct}=\left({\mathrm{Ct}}_{\mathrm{pvmdr}1}-{\mathrm{Ct}}_{\mathrm{pvaldolase}}\right)\\ \;\mathrm{sample}-\left({\mathrm{Ct}}_{\mathrm{pvmdr}1}-{\mathrm{Ct}}_{\mathrm{pvaldolase}}\right)\;\mathrm{calibrator} \end{array}$$

## Results

Of 801 malaria-positive isolates by microscopy, PCR analysis revealed that 536 (76%) isolates were positive for *P. vivax*, 128 (18%) were *P. falciparum*, and 43 (4%) were mixed infections of both *P. falciparum* plus *P. vivax*. None of the samples was positive for *P. malariae* or *P. ovale* and 94 samples were PCR-negative for *Plasmodium* DNA. Results discordant with microscopic diagnosis were re-tested by PCR for confirmation, and a further comparison of microscopy and PCR results is presented in a separate manuscript [42]. Age among the 801 subjects ranged from two months to 75 years, with a median of 24 years. 64% of samples were collected from males and 36% from females. The gender distribution was identical and age distribution nearly identical in the subset of 372 of 579 *P. vivax*-positive samples by PCR that were selected for sequencing (age range = two months to 70 years; median = 25 years). Of these, sequencing was successful in 90% (336/372) for *pvdhfr*, 91% (339/372) for *pvdhps* and 92% (342/372) for *pvmdr1*. A subset of 120 PCR-positive *P. vivax* isolates was randomly selected for the quantification of *pvmdr1* copy number variation, and the assay was successful in 108 (90%) of isolates.

Sequenced regions of each gene were monoclonal with no evidence of mixtures of SNPs among any of the samples, allowing the investigators to identify haplotypes. For codons I13L, P33L, F57L/I, S58R, T61M, S117N/T and I173L/F of *pvdhfr*, 151 (45%) of the isolates had the wild-type haplotype IPFSTSI. One hundred isolates (30%) had the S117N single mutation, 76 (23%) harboured S58R plus S117N double mutants, five isolates had the F57L plus S58R double mutant and one sample had the S58I plus S117N double mutant. The two mutations responsible for conferring pyrimethamine resistance *in vitro*, S58R and S117N, [46], were found in 84 (25%) and 177 (53%) of isolates, respectively. The *pvdhfr* S58R plus S117N double mutant haplotype was most prevalent among isolates in Sindh (6.8%) and Khyber Pakhtunkhwa province (6.5%) (Table 2). Although the *pvdhfr* double mutant was more common in infected patients over the age of 12 years than in children aged 12 and under prevalence of 24% versus 15%, the difference was not statistically significant (chi-square = 1.87; p = 0.17). Similarly, the double mutant was present in 28% of infections in females and 20% of infections in males but the difference was not significant (chi-square = 2.67; p = 0.10). Eight additional non-synonymous SNPs were detected, corresponding to amino acids N50I, S57R, S58R, S58I, S93R, S93H, S117N and N123K. Two of these, S93R and K123N, have not been reported previously. Non-synonymous and synonymous mutations are given in tabulated form in Additional file 1.

**Table 2:** **Prevalence and distribution of *pvdhfr*, *pvdhps *and *pvmdr1 *alleles by province**

	*pvdhfr		***pvdhps***		***pvmdr1***	
	**Haplotypes**	**No of isolates (%)**	**Haplotypes**	**No of isolates (%)**	**Haplotypes**	**No of isolates (%)**
**Balochistan**	IPFSTSI (wild type)	19 (5.7)	SAKAV (wild type)	44 (13)	YF (wild type)	2 (0.6)
	IPFST**N**I	18 (5.4)	S**G**KAV	1 (0.3)	Y**L**	38 (11)
	IPF**R**T**N**I	6 (2)				
**Total sequenced**	**43/50**		**45/50**		**40/50**	
**Islamabad**	IPFSTSI	26 (7.7)	SAKAV	47 (14)	YF	3 (1.0)
	IPF**R**T**N**I	14 (4.2)			Y**L**	47 (14)
	IPFST**N**I	10 (3.0)				
**Total sequenced**	**50/50**		**47/50**		**50/50**	
**Khyber Pakhtunkhwa**	IPFSTSI	37 (11)	SAKAV	108 (32)	YF	5 (2.0)
	IPFST**N**I	50 (15)	SAK**G**V	1 (0.3)	Y**L**	110 (32)
	IPF**R**T**N**I	22 (6.5)	S**G**KAV	4 (1.2)		
	IPF**R**TSI	2 (0.6)				
	IP**LR**TSI	1 (0.3)				
**Total sequenced**	**112/119**		**113/119**		**115/119**	
**Punjab**	IPFSTSI	55 (16)	SAKAV	89 (26)	YF	5 (2.0)
	IPF**R**T**N**I	12 (3.6)	S**G**KAV	1 (0.3)	Y**L**	84 (25)
	IPFST**N**I	12 (3.6)	S**G**K**G**V	1 (0.3)		
	IP**LR**TSI	4 (1.2)				
	IPF**R**TSI	1 (0.3)				
**Total sequenced**	**84/103**		**91/103**		**89/103**	
**Sindh**	IPFSTSI	14 (4.2)	SAKAV	42 (12)	YF	2 (0.6)
	IPF**R**T**N**I	23 (6.8)	S**G**KAV	1 (0.3)	Y**L**	46 (14)
	IPFST**N**I	10 (3.0)				
**Total sequenced**	**47/50**		**43/50**		**48/50**	

* Drug resistance-conferring point mutations are shown for the following codons: *pvdhfr* 13, 33, 57, 58, 61, 117, 173; *pvdhps* 382, 383, 512, 553, 585; and *pvmdr1* 976, 1076. Mutated amino acids are shown in bold.

Based on the use of *P. vivax* strain ARI*/*Pakistan (GenBank accession no X98123) as reference for *pvdhfr*, three indels were detected in the study, all 18 base pairs (bp) in size (Table 3). Seven isolates had an 18 bp repeat insertion between amino acid positions 91 and 92, and 21 samples had an 18 bp repeat insertion between codons 103 and 104. Ten isolates were discovered with an 18-bp deletion between amino acids 92 and 97.

**Table 3:** **Insertions and deletions in *pvdhfr *gene of *Plasmodium vivax***

**Insertions/Deletions**	**Codons**	**No of bp**	**Sequence**	**No of samples**
Insertion A	91,92	18 bp	ACACACGGTGGTGACAAC	7
Insertion B	103,104	18 bp	ACACACGGTGGTGACAAT	29
Deletion	92,97	18 bp	AGCGGTGGTGACAACACA	10

The majority of samples were wild-type for *pvdhps* point mutations associated with sulphadoxine resistance, with the exception of seven samples carrying the A383G mutation, one carrying the A553G mutation, and one carrying the A383G plus A553G double mutation (Table 1). Four of the samples carrying the A383G mutation were from Khyber Pakhtunkhwa while the other three were found in each of three provinces, and the A383G plus A553G double mutant was found in Punjab (Table 2). Six non-synonymous mutations were detected (Additional file 1), four of which have not been previously reported.

The three most prevalent *pvdhfr* plus *pvdhps* haplotypes across all sites were wild-type (44%), single *pvdhfr* mutantI_13_P_33_F_57_S_58_T_61_**N**_117_I_173_/ S_382_A_383_K_512_A_553_V_585_ (30%) and double *pvdhfr* mutant I_13_P_33_F_57_**R**_58_T_61_**N**_117_I_173_/ S_382_A_383_K_512_A_553_V_585_ (21%).

All isolates were wild type at *pvmdr1* codon Y976F while 335 (98%) isolates carried the point mutation at codon F1076L (Table 1). One hundred and ten, or 32% of all isolates with *pvmdr1* F1076L, were found in Khyber Pakhtunkhwa and 84 (25%) were found in Punjab province. Three other non-synonymous SNPs (Y963C, N1010S and S1071G) not previously reported and a single synonymous SNP were also found (Additional file 1). All 108 isolates tested carried a single copy of the *pvmdr1* gene.

## Discussion

Pakistan adopted artesunate plus SP combination therapy as a first line treatment against uncomplicated *P. falciparum* in 2008 [47]. Chloroquine is no longer indicated for treatment of *P. falciparum* but is recommended in combination with primaquine for treatment of *P. vivax*. Although the nature of anti-malarial use in Pakistan is not well-characterized, the availability of SP and other drugs as monotherapy has been documented [48] and, along with misdiagnosis of mixed infections [49] and presumptive treatment [50], likely results in *P. vivax* infections being frequently treated with AS + SP or SP alone. This study sought to characterize the current distribution of chloroquine resistance-associated polymorphisms in *pvmdr1* and SP resistance-associated point mutations in *pvdhfr* and *pvdhps* in Pakistan.

Resistance of *P. falciparum* to chloroquine is nearly fixed in Pakistan [3,51], and the emergence of chloroquine-resistant *P. vivax* in neighbouring India [52-54] along with established resistance in Thailand [55,56], Indonesia [57,58] and Vietnam [59] suggests that the treatment of *P. vivax* with chloroquine in Pakistan could be in danger of being compromised. Clinical trials conducted during 2004-2006 indicate that chloroquine was still efficacious against *P. vivax* in Pakistan [9]. As a more recent follow-on to clinical evidence, this study presents the first report of *pvmdr1* gene polymorphisms in Pakistan. Nearly all isolates were wild type at *pvmdr1* Y796F, suggesting that chloroquine resistance in *P. vivax* has not emerged, but many carried the F1076L mutant. These results are consistent with previous studies showing the rise of *pvmdr1* F1076L prior to Y976F [12,23,60]. If the hypothesis that the two-step trajectory of mutations at codon F1076L followed by Y976F may lead to chloroquine resistance [12] is correct, then *pvmdr1* F1076L may provide an early signal of emerging chloroquine resistance in Pakistan prior to the appearance of the drug resistance phenotype in the population [22], particularly in Khyber Pakhtunkhwa where its prevalence is highest. Polymorphisms in *pvmdr1* should continue to be monitored in case the mutation at position Y976F appears. Amplification of the *pvmdr1* gene was not detected in this study, reflecting the absence of mefloquine drug pressure in Pakistan. Regions with a history of mefloquine use for *falciparum* malaria have shown amplification of both *pfmdr1* in *P. falciparum* and *pvmdr1* in *P. vivax*, likely caused by inadvertent drug exposure [17,23].

*Plasmodium vivax* is likely exposed to SP pressure (either alone or in combination with artesunate) in a bystander effect, particularly because it is found alongside *P. falciparum* in regions with mixed-species infections such as Balochistan (10%), Khyber Pakhtunkhwa (8%), and Punjab (5%) [42], that are vulnerable to misdiagnosis of *Plasmodium* species [3,41,49,51]. Exposure of *P. vivax* parasites to SP pressure, with likely subtherapeutic levels in some patients, could favour the emergence of SP resistance [61].

In this study, the *pvdhfr* double mutant S58R and S117N was found in 20% of isolates. The double mutant has been reported previously from one site in Pakistan [3] as well as in neighbouring India [62] and Iran [63], and is associated with *in vitro* pyrimethamine resistance [64]. In contrast, point mutations in *pvdhps* A383G and A553G were detected in a very small number of samples, confirming similar results from a previous report in Bannu district in 2007 [3]. These mutations have also been reported as rare in Iran [63] and Afghanistan [65], suggesting that markers associated with sulphadoxine resistance in *P. vivax* remain rare in this region. Because SP treatment failure is closely associated with infections carrying four or more mutations in *pvdhfr*[35] and additional *pvdhps* mutations [66], it appears that SP likely remains efficacious against *P. vivax* in Pakistan as previously reported [9].

The prevalence of *pvdhfr* S117N single and S58R plus S117N double mutants was highest in Sindh and Khyber Pakhtunkhwa provinces. Khyber Pakhtunkhwa had the second highest prevalence of mixed species and *P. falciparum* infections in this survey [42], indicating that perhaps regions with *P. falciparum* experience higher SP use (with or without artesunate) that also exerts pressure on co-occurring *P. vivax* infections. Khyber Pakhtunkhwa province also had the highest prevalence of *pvmdr1* F1076L mutants, and may be especially vulnerable to the influx or emergence of drug-resistant parasites because of the large-scale migration of Afghan refugees and internally displaced people (IDPs) from conflict areas to this region, along with conditions of underdevelopment that may prevent adequate diagnosis and treatment of malaria [67].

## Conclusions

This is the first comprehensive report on molecular patterns of drug resistance in *P. vivax* in Pakistan. The major chloroquine resistance-mediating mutation was not detected in this study; however continued molecular monitoring of polymorphisms in *pvmdr1* is crucial to detect the potential emergence of chloroquine-resistant *P. vivax* in Pakistan. Although some evidence of pyrimethamine selective pressure was observed, polymorphisms associated with sulphadoxine resistance were rare, and markers of clinically relevant SP resistance in *P. vivax* were not present. Thus, the data on molecular markers presented here suggest that efficacy of both chloroquine and SP against *P. vivax* is currently high, and can serve as a baseline reference for regional studies of *P. vivax* drug sensitivity in Pakistan.

## Competing interests

The authors declare that they have no competing interests.

## Authors’ contributions

AAK designed study, carried out the laboratory experiments, and performed data analysis. AAK and MV drafted the manuscript. AO and LJK contributed to laboratory experiments and sequence analysis. MFN and FN participated in sample collection and microscopy. MV, LK, SAM and CVP provided guidance and coordination for study design, genotyping, and data analyses, and edited and revised the manuscript. All authors read and approved the final manuscript.

## Supplementary Material

Additional file 1 **Synonymous and non-synonymous SNPs detected in *pvdhfr, pvdhps *and *pvmdr1.*** Click here for file

## References

[B1] WHO World malaria report 2012 2013 Geneva: World Health Organization

[B2] D MC Malaria No More 2013 Islamabad: Directorate of Malaria Control, Ministry of National Health Services

[B3] Khatoon L Baliraine FN Bonizzoni M Malik SA Yan G Prevalence of antimalarial drug resistance mutations in *Plasmodium vivax* and *P. falciparum* from a malaria-endemic area of Pakistan Am J Trop Med Hyg 2009 81 525 528 19706926 PMC2789568

[B4] Rieckmann KH Davis DR Hutton DC *Plasmodium vivax* resistance to chloroquine? Lancet 1989 2 1183 1184 2572903 10.1016/s0140-6736(89)91792-3

[B5] Soto J Toledo J Gutierrez P Luzz M Llinas N Cedeno N Dunne M Berman J *Plasmodium vivax* clinically resistant to chloroquine in Colombia Am J Trop Med Hyg 2001 65 90 93 11508397 10.4269/ajtmh.2001.65.90

[B6] Kurcer MA Simsek Z Kurcer Z The decreasing efficacy of chloroquine in the treatment of *Plasmodium vivax* malaria, in Sanliurfa, south-eastern Turkey Ann Trop Med Parasitol 2006 100 109 113 10.1179/136485906X86284 16492358

[B7] Phan GT de Vries PJ Tran BQ Le HQ Nguyen NV Nguyen TV Heisterkamp SH Kager PA Artemisinin or chloroquine for blood stage *Plasmodium vivax* malaria in Vietnam Trop Med Int Health 2002 7 858 864 10.1046/j.1365-3156.2002.00948.x 12358621

[B8] Awab GR Pukrittayakamee S Imwong M Dondorp AM Woodrow CJ Lee SJ Day NP Singhasivanon P White NJ Kaker F Dihydroartemisinin-piperaquine versus chloroquine to treat *vivax* malaria in Afghanistan: an open randomized, non-inferiority, trial Malar J 2010 9 105 10.1186/1475-2875-9-105 20409302 PMC2864284

[B9] Leslie T Mayan MI Hasan MA Safi MH Klinkenberg E Whitty CJ Rowland M Sulfadoxine-pyrimethamine, chlorproguanil-dapsone, or chloroquine for the treatment of *Plasmodium vivax* malaria in Afghanistan and Pakistan: a randomized controlled trial JAMA 2007 297 2201 2209 10.1001/jama.297.20.2201 17519409

[B10] Valecha N Joshi H Eapen A Ravinderan J Kumar A Prajapati SK Ringwald P Therapeutic efficacy of chloroquine in *Plasmodium vivax* from areas with different epidemiological patterns in India and their *Pvdhfr* gene mutation pattern Trans R Soc Trop Med Hyg 2006 100 831 837 10.1016/j.trstmh.2005.11.012 16513151

[B11] Nandy A Addy M Maji AK Bandyopadhyay AK Monitoring the chloroquine sensitivity of *Plasmodium vivax* from Calcutta and Orissa, India Ann Trop Med Parasitol 2003 97 215 220 10.1179/000349803235001868 12803853

[B12] Brega S Meslin B de Monbrison F Severini C Gradoni L Udomsangpetch R Sutanto I Peyron F Picot S Identification of the *Plasmodium vivax mdr*-like gene (*pvmdr1*) and analysis of single-nucleotide polymorphisms among isolates from different areas of endemicity J Infect Dis 2005 191 272 277 10.1086/426830 15609238

[B13] Nomura T Carlton JM Baird JK Del Portillo HA Fryauff DJ Rathore D Fidock DA Su X Collins WE McCutchan TF Wootton JC Wellems TE Evidence for different mechanisms of chloroquine resistance in 2 *Plasmodium* species that cause human malaria J Infect Dis 2001 183 1653 1661 10.1086/320707 11343215

[B14] Sa JM Nomura T Neves J Baird JK Wellems TE Del Portillo HA *Plasmodium vivax*: allele variants of the mdr1 gene do not associate with chloroquine resistance among isolates from Brazil, Papua, and monkey-adapted strains Exp Parasitol 2005 109 256 259 10.1016/j.exppara.2004.12.005 15755424

[B15] Marfurt J de Monbrison F Brega S Barbollat L Muller I Sie A Goroti M Reeder JC Beck HP Picot S Genton B Molecular markers of *in vivo Plasmodium vivax* resistance to amodiaquine plus sulfadoxine-pyrimethamine: mutations in *pvdhfr* and *pvmdr1* J Infect Dis 2008 198 409 417 10.1086/589882 18582193

[B16] Suwanarusk R Russell B Chavchich M Chalfein F Kenangalem E Kosaisavee V Prasetyorini B Piera KA Barends M Brockman A Lek-Uthai U Anstey NM Tjitra E Nosten F Cheng Q Price RN Chloroquine resistant *Plasmodium vivax*: *in vitro* characterisation and association with molecular polymorphisms PLoS One 2007 2 e1089 10.1371/journal.pone.0001089 17971853 PMC2034531

[B17] Imwong M Pukrittayakamee S Pongtavornpinyo W Nakeesathit S Nair S Newton P Nosten F Anderson TJ Dondorp A Day NP White NJ Gene amplification of the multidrug resistance 1 gene of *Plasmodium vivax* isolates from Thailand, Laos, and Myanmar Antimicrob Agents Chemother 2008 52 2657 2659 10.1128/AAC.01459-07 18443118 PMC2443893

[B18] Lu F Wang B Cao J Sattabongkot J Zhou H Zhu G Kim K Gao Q Han ET Prevalence of drug resistance-associated gene mutations in *Plasmodium vivax* in Central China Korean J Parasitol 2012 50 379 384 10.3347/kjp.2012.50.4.379 23230341 PMC3514435

[B19] Mint LK Ould Mohamed Salem BA Gaillard T Wurtz N Bogreau H Hafid JE Trape JF Bouchiba H Ould Ahmedou Salem MS Pradines B Rogier C Basco LK Briolant S Molecular surveillance of drug-resistant *Plasmodium vivax* using *pvdhfr*, *pvdhps* and *pvmdr1* markers in Nouakchott, Mauritania J Antimicrob Chemother 2012 67 367 374 10.1093/jac/dkr464 22086859

[B20] Ranjitkar S Schousboe ML Thomsen TT Adhikari M Kapel CM Bygbjerg IC Alifrangis M Prevalence of molecular markers of anti-malarial drug resistance in *Plasmodium vivax* and *Plasmodium falciparum* in two districts of Nepal Malar J 2011 10 75 10.1186/1475-2875-10-75 21457533 PMC3080351

[B21] Jovel IT Mejia RE Banegas E Piedade R Alger J Fontecha G Ferreira PE Veiga MI Enamorado IG Bjorkman A Ursing J Drug resistance associated genetic polymorphisms in *Plasmodium falciparum* and *Plasmodium vivax* collected in Honduras Central America. Malar J 2011 10 376 10.1186/1475-2875-10-376 PMC3266654 22183028

[B22] Orjuela-Sanchez P Karunaweera ND da Silva-Nunes M da Silva NS Scopel KK Goncalves RM Amaratunga C Sa JM Socheat D Fairhust RM Gunawardena S Thavakodirasah T Galapaththy GL Abeysinghe R Kawamoto F Wirth DF Ferreira MU Single-nucleotide polymorphism, linkage disequilibrium and geographic structure in the malaria parasite *Plasmodium vivax*: prospects for genome-wide association studies BMC Genet 2010 11 65 20626846 10.1186/1471-2156-11-65 PMC2910014

[B23] Suwanarusk R Chavchich M Russell B Jaidee A Chalfein F Barends M Prasetyorini B Kenangalem E Piera KA Lek-Uthai U Anstey NM Tjitra E Nosten F Cheng Q Price RN Amplification of *pvmdr1* associated with multidrug-resistant *Plasmodium vivax* J Infect Dis 2008 198 1558 1564 10.1086/592451 18808339 PMC4337975

[B24] Alam MT Bora H Bharti PK Saifi MA Das MK Dev V Kumar A Singh N Dash AP Das B Wajihullah Sharma YD Similar trends of pyrimethamine resistance-associated mutations in *Plasmodium vivax* and *P. falciparum* Antimicrob Agents Chemother 2007 51 857 863 10.1128/AAC.01200-06 17194833 PMC1803105

[B25] Hastings MD Porter KM Maguire JD Susanti I Kania W Bangs MJ Sibley CH Baird JK Dihydrofolate reductase mutations in *Plasmodium vivax* from Indonesia and therapeutic response to sulfadoxine plus pyrimethamine J Infect Dis 2004 189 744 750 10.1086/381397 14767830

[B26] Imwong M Pukrittayakamee S Renia L Letourneur F Charlieu JP Leartsakulpanich U Looareesuwan S White NJ Snounou G Novel point mutations in the dihydrofolate reductase gene of *Plasmodium vivax*: evidence for sequential selection by drug pressure Antimicrob Agents Chemother 2003 47 1514 1521 10.1128/AAC.47.5.1514-1521.2003 12709316 PMC153323

[B27] Wang P Read M Sims PF Hyde JE Sulfadoxine resistance in the human malaria parasite *Plasmodium falciparum* is determined by mutations in dihydropteroate synthetase and an additional factor associated with folate utilization Mol Microbiol 1997 23 979 986 10.1046/j.1365-2958.1997.2821646.x 9076734

[B28] Triglia T Cowman AF Primary structure and expression of the dihydropteroate synthetase gene of *Plasmodium falciparum* Proc Natl Acad Sci USA 1994 91 7149 7153 10.1073/pnas.91.15.7149 8041761 PMC44356

[B29] Triglia T Menting JG Wilson C Cowman AF Mutations in dihydropteroate synthase are responsible for sulfone and sulfonamide resistance in *Plasmodium falciparum* Proc Natl Acad Sci USA 1997 94 13944 13949 10.1073/pnas.94.25.13944 9391132 PMC28412

[B30] Peterson DS Walliker D Wellems TE Evidence that a point mutation in dihydrofolate reductase-thymidylate synthase confers resistance to pyrimethamine in *falciparum* malaria Proc Natl Acad Sci USA 1988 85 9114 9118 10.1073/pnas.85.23.9114 2904149 PMC282674

[B31] Peterson DS Milhous WK Wellems TE Molecular basis of differential resistance to cycloguanil and pyrimethamine in *Plasmodium falciparum* malaria Proc Natl Acad Sci USA 1990 87 3018 3022 10.1073/pnas.87.8.3018 2183222 PMC53825

[B32] Foote SJ Galatis D Cowman AF Amino acids in the dihydrofolate reductase-thymidylate synthase gene of *Plasmodium falciparum* involved in cycloguanil resistance differ from those involved in pyrimethamine resistance Proc Natl Acad Sci USA 1990 87 3014 3017 10.1073/pnas.87.8.3014 2183221 PMC53824

[B33] Cowman AF Morry MJ Biggs BA Cross GA Foote SJ Amino acid changes linked to pyrimethamine resistance in the dihydrofolate reductase-thymidylate synthase gene of *Plasmodium falciparum* Proc Natl Acad Sci USA 1988 85 9109 9113 10.1073/pnas.85.23.9109 3057499 PMC282673

[B34] Brooks DR Wang P Read M Watkins WM Sims PF Hyde JE Sequence variation of the hydroxymethyldihydropterin pyrophosphokinase: dihydropteroate synthase gene in lines of the human malaria parasite, *Plasmodium falciparum*, with differing resistance to sulfadoxine Eur J Biochem 1994 224 397 405 10.1111/j.1432-1033.1994.00397.x 7925353

[B35] Hawkins VN Joshi H Rungsihirunrat K Na-Bangchang K Sibley CH Antifolates can have a role in the treatment of *Plasmodium vivax* Trends Parasitol 2007 23 213 222 10.1016/j.pt.2007.03.002 17368986

[B36] Auliff A Wilson DW Russell B Gao Q Chen N Anh LN Maguire J Bell D O’Neil MT Cheng Q Amino acid mutations in *Plasmodium vivax DHFR* and *DHPS* from several geographical regions and susceptibility to antifolate drugs Am J Trop Med Hyg 2006 75 617 621 17038682

[B37] Hastings MD Sibley CH Pyrimethamine and WR99210 exert opposing selection on dihydrofolate reductase from *Plasmodium vivax* Proc Natl Acad Sci USA 2002 99 13137 13141 10.1073/pnas.182295999 12198181 PMC130599

[B38] Hastings MD Maguire JD Bangs MJ Zimmerman PA Reeder JC Baird JK Sibley CH Novel *Plasmodium vivax dhfr* alleles from the Indonesian Archipelago and Papua New Guinea: association with pyrimethamine resistance determined by a Saccharomyces cerevisiae expression system Antimicrob Agents Chemother 2005 49 733 740 10.1128/AAC.49.2.733-740.2005 15673758 PMC547327

[B39] Leartsakulpanich U Imwong M Pukrittayakamee S White NJ Snounou G Sirawaraporn W Yuthavong Y Molecular characterization of dihydrofolate reductase in relation to antifolate resistance in *Plasmodium vivax* Mol Biochem Parasitol 2002 119 63 73 10.1016/S0166-6851(01)00402-9 11755187

[B40] Triglia T Wang P Sims PF Hyde JE Cowman AF Allelic exchange at the endogenous genomic locus in *Plasmodium falciparum* proves the role of dihydropteroate synthase in sulfadoxine-resistant malaria EMBO J 1998 17 3807 3815 10.1093/emboj/17.14.3807 9669998 PMC1170716

[B41] Zakeri S Afsharpad M Ghasemi F Raeisi A Kakar Q Atta H Djadid ND *Plasmodium vivax*: prevalence of mutations associated with sulfadoxine-pyrimethamine resistance in *Plasmodium vivax* clinical isolates from Pakistan Exp Parasitol 2011 127 167 172 10.1016/j.exppara.2010.07.011 20655913

[B42] Khattak AA Venkatesan M Nadeem MF Satti HS Yaqoob Strauss Khatoon Malik S Plowe CV Prevalence and distribution of human *Plasmodium* infection in Pakistan Malar J 2013 12 1 297 [Epub ahead of print] PubMed PMID: 23984968. 10.1186/1475-2875-12-297 23984968 PMC3765785

[B43] Nawaz S FATA—A Most Dangerous Place 2009 Washington, D.C: Center for Strategic and International Studies

[B44] Asif SA Departmental audit of malaria control programme 2001-2005 north west frontier province (NWFP) J Ayub Med Coll Abbottabad 2008 20 98 102 19024199

[B45] Snounou G Viriyakosol S Jarra W Thaithong S Brown KN Identification of the four human malaria parasite species in field samples by the polymerase chain reaction and detection of a high prevalence of mixed infections Mol Biochem Parasitol 1993 58 283 292 10.1016/0166-6851(93)90050-8 8479452

[B46] Tjitra E Baker J Suprianto S Cheng Q Anstey NM Therapeutic efficacies of artesunate-sulfadoxine-pyrimethamine and chloroquine-sulfadoxine-pyrimethamine in *vivax* malaria pilot studies: relationship to *Plasmodium vivax dhfr* mutations Antimicrob Agents Chemother 2002 46 3947 3953 10.1128/AAC.46.12.3947-3953.2002 12435700 PMC132782

[B47] WHO World Malaria Report 2008 2009 Geneva: World Health Organization

[B48] Khan SY Khan A Arshad M Tahir HM Mukhtar MK Ahmad KR Arshad N Irrational use of antimalarial drugs in rural areas of eastern Pakistan: a random field study BMC Publ Health 2012 12 941 10.1186/1471-2458-12-941 PMC3577451 23116148

[B49] Zakeri S Kakar Q Ghasemi F Raeisi A Butt W Safi N Afsharpad M Memon MS Gholizadeh S Salehi M Atta H Zamani G Djadid ND Detection of mixed *Plasmodium falciparum* &*P*. *vivax* infections by nested-PCR in Pakistan, Iran & Afghanistan Indian J Med Res 2010 132 31 35 20693586

[B50] Malik M Hassali MAA Shafie AA Hussain A Why Don't Medical Practitioners Treat Malaria Rationally? A Qualitative Study from Pakistan Trop J Pharm Res 2012 4 673 681

[B51] Ghanchi NK Ursing J Beg MA Veiga MI Jafri S Martensson A Prevalence of resistance associated polymorphisms in *Plasmodium falciparum* field isolates from southern Pakistan Malar J 2011 10 18 10.1186/1475-2875-10-18 21272384 PMC3037930

[B52] Dua VK Kar PK Sharma VP Chloroquine resistant *Plasmodium vivax* malaria in India Trop Med Int Health 1996 1 816 819 8980595 10.1111/j.1365-3156.1996.tb00116.x

[B53] Garg M Gopinathan N Bodhe P Kshirsagar NA *Vivax* malaria resistant to chloroquine: case reports from Bombay Trans R Soc Trop Med Hyg 1995 89 656 657 10.1016/0035-9203(95)90432-8 8594687

[B54] Van den Abbeele K Van den Enden E Van den Ende J Combined chloroquine and primaquine resistant *Plasmodium vivax* malaria in a patient returning from India Ann Soc Belg Med Trop 1995 75 73 74 7794065

[B55] Congpuong K Na-Bangchang K Thimasarn K Tasanor U Wernsdorfer WH Sensitivity of *Plasmodium vivax* to chloroquine in Sa Kaeo Province, Thailand Acta Trop 2002 83 117 121 10.1016/S0001-706X(02)00090-6 12088852

[B56] Looareesuwan S Buchachart K Wilairatana P Chalermrut K Rattanapong Y Amradee S Siripiphat S Chullawichit S Thimasan K Ittiverakul M Triampon A Walsh DS Primaquine-tolerant *vivax* malaria in Thailand Ann Trop Med Parasitol 1997 91 939 943 9579214 10.1080/00034989760338

[B57] Schwartz IK Lackritz EM Patchen LC Chloroquine-resistant *Plasmodium vivax* from Indonesia N Engl J Med 1991 324 927 2000121 10.1056/NEJM199103283241317

[B58] Collignon P Chloroquine resistance in *Plasmodium vivax* J Infect Dis 1991 164 222 223 10.1093/infdis/164.1.222 2056216

[B59] Taylor WR Doan HN Nguyen DT Tran TU Fryauff DJ Gomez-Saladin E Kain KC Le DC Baird JK Assessing drug sensitivity of *Plasmodium vivax* to halofantrine or choroquine in southern, central Vietnam using an extended 28-day *in vivo* test and polymerase chain reaction genotyping Am J Trop Med Hyg 2000 62 693 697 11304056 10.4269/ajtmh.2000.62.693

[B60] Orjuela-Sanchez P de Santana Filho FS Machado-Lima A Chehuan YF Costa MR Alecrim M Del Portillo HA Analysis of single-nucleotide polymorphisms in the *crt-o* and *mdr1* genes of *Plasmodium vivax* among chloroquine-resistant isolates from the Brazilian Amazon region Antimicrob Agents Chemother 2009 53 3561 3564 10.1128/AAC.00004-09 19451296 PMC2715622

[B61] Hastings IM Watkins WM Tolerance is the key to understanding antimalarial drug resistance Trends Parasitol 2006 22 71 77 10.1016/j.pt.2005.12.011 16406706

[B62] Prajapati SK Joshi H Dev V Dua VK Molecular epidemiology of *Plasmodium vivax* anti-folate resistance in India Malar J 2011 10 102 10.1186/1475-2875-10-102 21513569 PMC3098820

[B63] Zakeri S Motmaen SR Afsharpad M Djadid ND Molecular characterization of antifolates resistance-associated genes, (*dhfr* and *dhps*) in *Plasmodium vivax* isolates from the Middle East Malar J 2009 8 20 10.1186/1475-2875-8-20 19175936 PMC2653067

[B64] Imwong M Pukrittakayamee S Looareesuwan S Pasvol G Poirreiz J White NJ Snounou G Association of genetic mutations in *Plasmodium vivax dhfr* with resistance to sulfadoxine-pyrimethamine: geographical and clinical correlates Antimicrob Agents Chemother 2001 45 3122 3127 10.1128/AAC.45.11.3122-3127.2001 11600366 PMC90792

[B65] Zakeri S Afsharpad M Ghasemi F Raeisi A Safi N Butt W Atta H Djadid ND Molecular surveillance of *Plasmodium vivax dhfr* and *dhps* mutations in isolates from Afghanistan Malar J 2010 9 75 10.1186/1475-2875-9-75 20226087 PMC2848684

[B66] Imwong M Pukrittayakamee S Cheng Q Moore C Looareesuwan S Snounou G White NJ Day NP Limited polymorphism in the dihydropteroate synthetase gene (*dhps*) of *Plasmodium vivax* isolates from Thailand Antimicrob Agents Chemother 2005 49 4393 4395 10.1128/AAC.49.10.4393-4395.2005 16189131 PMC1251524

[B67] Department of Health Government of Khyber Pakhtunkhwa Brief of roll back malaria programme Khyber Pakhtunkhwa 2013 1 3 http://www.healthkp.gov.pk/downloads/rbm.doc

