# Supplementary material for: Prevalence and patterns of antifolate and chloroquine drug resistance markers in Plasmodium vivax across Pakistan
Source: Malar J. 2013 Sep 5;12:310. doi: 10.1186/1475-2875-12-310 (PMC3766695; doi:10.1186/1475-2875-12-310)
Supplement: Additional file 1 — Synonymous and non-synonymous SNPs detected in pvdhfr, pvdhps and pvmdr1. [file 1475-2875-12-310-S1.doc]

**Additional file 1.**  **Synonymous and non-synonymous SNPs detected in *pvdhfr*, *pvdhps* and *pvmdr1***

| **Non-Synonymous mutations** | | | |
| --- | --- | --- | --- |
| **Nucleotide substitution** | **Amino acid substitution** | **No of isolates** | **Prevalence (%)** |
| ***Pvdhfr*** | | | |
| 150: A→T | 50: N → I | 55 | 16.4 |
| 279: A→C | 93: S → R* | 24 | 7.1 |
| 279: G→A | 93: S → H | 1 | 0.3 |
| 369: G→C | 123: N → K* | 1 | 0.3 |
| ***Pvdhps*** | | | |
| 1095: T→C | 365: F → L | 8 | 2.4 |
| 1101:A→C | 367: M → L* | 11 | 3.2 |
| 1377: A→C | 459: D → A | 19 | 5.6 |
| 1494: T→C | 498: V → A* | 1 | 0.3 |
| 1662: A→C | 554: K → G* | 1 | 0.3 |
| 1710: G→C | 570: D → H* | 1 | 0.3 |
| ***Pvmdr1*** | | | |
| 2889: A→G | 963: Y→C* | 1 | 0.3 |
| 3030: A→G | 1010: N→S* | 5 | 1.5 |
| 3213: A→G | 1071: S→G* | 1 | 0.3 |
| **Synonymous mutations** | | | |
| ***Pvdhfr*** | | | |
| 45: A→G | A | 5 | 1.5 |
| 72: G→A | E | 1 | 0.3 |
| 114: G→C | G | 3 | 1 |
| 189: C→T | Y | 1 | 0.3 |
| 207: T→C | Y | 75 | 22 |
| 309: C→T | N | 2 | 0.6 |
| 369: G→A | N | 2 | 0.6 |
| 402: T→C | V | 7 | 2.1 |
| ***Pvdhps*** | | | |
| 463: G→A | E | 2 | 0.6 |
| 498: C→T | V | 1 | 0.3 |
| ***Pvmdr1*** | | | |
| 970: T→A | A | 1 | 0.3 |

*Not previously reported
